# Cost-effectiveness and benefit-risk of rotavirus vaccination in Afghanistan: a modelling analysis informed by post-licensure surveillance

**DOI:** 10.1186/s12913-025-12885-5

**Published:** 2025-07-04

**Authors:** Palwasha Anwari, Frédéric Debellut, Sardar Parwiz, Clint Pecenka, Andrew Clark

**Affiliations:** 1 London School of Hygiene and Tropical Medicine, Keppel Street, London, WC1E 7HT England, UK; 2 PATH, Rue De Varembe 7, Geneva, 1202 Switzerland; 3 EMPHNET, Abdullah Ben Abbas St 42, Amman, Jordan; 4 PATH, 2201 Westlake Ave, Suite 200, Seattle, WA 98121 USA

**Keywords:** Rotavirus vaccine, Cost-utility, Cost-effectiveness, Benefit-risk, Rotavirus gastroenteritis, Afghanistan

## Abstract

**Introduction:**

Afghanistan added ROTARIX to the routine national immunization programme in 2018. We aimed to estimate the cost-effectiveness and benefit-risk of ROTARIX and compare its continued use with other rotavirus vaccines that could be used in the future.

**Methods:**

We used a static cohort model with a finely disaggregated age structure (weeks of age < 5 years) to assess the use of ROTARIX (1-dose vial) over a seven-year period (2018–2024) in Afghanistan. The primary outcome measure was the discounted cost (2022 US$) per Disability Adjusted Life Year (DALY) averted (from government and societal perspectives) compared to no vaccination. We also calculated the benefit-risk ratio i.e., the number of RVGE deaths prevented per one excess intussusception death. Model inputs were informed by pre- and post-licensure surveillance data, new analyses of household survey data, and updated estimates from the international literature. We ran a separate analysis to compare the potential cost-effectiveness and benefit-risk of ROTARIX (1-dose vial), ROTASIIL (1-dose vial), ROTASIIL (2-dose vial), and ROTAVAC (5-dose vial) over a ten-year period (2025–2034). Each product was compared to no rotavirus vaccination and each other. We ran deterministic and probabilistic uncertainty analyses and interpreted our results over a range of cost-effectiveness thresholds.

**Findings:**

We estimated that routine use of ROTARIX between 2018 and 2024 has prevented 4,600 RVGE deaths (a 41% reduction), 86,400 hospital admissions, and 1.72 million RVGE cases. For every 1,493 RVGE deaths prevented by the vaccine, we estimated one potential excess intussusception death. With a heavily reduced vaccine dose cost (due to support from Gavi) the net cost to the Afghanistan government vaccine programme was estimated to be US$ 4.4 million per year. The cost per DALY averted was US$ 125 (0.25 times the national GDP per capita) when using a Gavi-subsidised vaccine cost and including household costs averted by vaccination. This increased to US$ 471 (0.94 times the national GDP per capita) when incorporating the full vaccine price without Gavi’s subsidy and excluding household costs averted by vaccination. When assuming continued Gavi support over the period 2025–2034, the dominant product would be ROTARIX. Without Gavi support, ROTASIIL (2-dose vial) dominates.

**Conclusion:**

Our study supports the sustained use of rotavirus vaccination in Afghanistan. The health benefits of the vaccine greatly exceed the potential risks.

**Supplementary Information:**

The online version contains supplementary material available at 10.1186/s12913-025-12885-5.

## Introduction

Acute gastroenteritis (AGE) continues to be a leading cause of mortality in children < 5 years old, accounting for over half a million deaths annually and 10% of all fatalities in this age group worldwide [1, 2]. Rotavirus gastroenteritis (RVGE) is estimated to be responsible for 24–37% of AGE deaths [3, 4]. Following the World Health Organisation (WHO) recommendation for universal rotavirus (RV) vaccination in 2009, more than 120 countries have incorporated the vaccine into their national immunization programmes [5, 6]. Gavi, the vaccine alliance, has played a pivotal role by offering financial assistance to facilitate the introduction of the RV vaccine in low-income countries [6]. At the global level, RVGE deaths in children aged < 5 years have decreased from around 450,000 in 2008 to around 150,000 in 2019 [3, 7, 8]. Recent estimates suggest that RV vaccines prevented 139,000 RVGE deaths in children < 5 years old from 2006 to 2019 [4]. RVGE also imposes an important economic burden; a cost-effectiveness study of 73 Gavi-eligible countries indicated that RV vaccines could avert an estimated US$ 878 million in healthcare costs from the societal perspective over the period 2018–2027 [9].

In January 2018, Afghanistan added ROTARIX (GlaxoSmithKline, Belgium), one of the two pre-qualified rotavirus vaccines available at that time, to the national routine immunization programme, with financial support from Gavi. ROTARIX is an oral vaccine administered to infants in a two-dose schedule at 6 and 10 weeks of age [10]. The decision to introduce the vaccine was informed by a modelling study led by members of the Afghanistan National Immunization Technical Advisory Group (NITAG) and the Ministry of Public Health (MoPH). This study estimated that ROTARIX could prevent 25% of RVGE deaths aged < 5 years at a cost of < US$ 100 per DALY averted [11].

Following the introduction of ROTARIX, post-licensure surveillance was established at five sites (Indira Gandhi Children Hospital, Herat Regional Hospital, Nangarhar Regional Hospital, Mazar Regional Hospital, and Ataturk Children Hospital) to monitor the effectiveness and safety of ROTARIX. Moderate vaccine effectiveness (VE) (45%; 95% CI: 22, 62) was demonstrated against RVGE hospital admissions aged 6–59 months old, and the overall vaccine impact (percent reduction in RVGE admissions aged < 5 years) was 39% [12]. Importantly, no substantial increased risk of intussusception (a rare type of bowel blockage) was observed except a possible increase in risk of intussusception 8–21 days after receiving the first dose [13, 14].

There are several reasons why the original economic evaluation of rotavirus vaccination in Afghanistan should be updated. First, the collapse of the Afghan government in mid-August 2021 disrupted financial support from international donors, which had previously played a crucial role in sustaining the healthcare system. While international financial aid resumed in October 2021, it has been significantly reprioritized, with a primary focus on humanitarian response rather than long-term health system strengthening. Routine immunization programmes, including rotavirus vaccination, continue to receive funding; however, budget constraints have intensified, requiring stronger justification for continued investment in vaccine programmes. This situation underscores the critical need to reassess the cost-effectiveness of rotavirus vaccination within the current fiscal landscape, ensuring that immunization remains a priority in an increasingly resource-limited setting. Second, post-licensure surveillance data on the real-world effectiveness of ROTARIX are now available, together with updated estimates of pre-vaccination rotavirus mortality, rotavirus vaccine coverage, and vaccine timeliness. Third, new post-licensure data on vaccine safety provides an opportunity to assess benefit-risk. Finally, four rotavirus vaccines are now available, and an updated analysis provides an opportunity to compare the cost-effectiveness of other available products on the global market [15]. ROTARIX is administered as a two-dose course, while the other three products (ROTATEQ [Merck & Co., USA], ROTASIIL [Serum Institute of India Pvt. Ltd. India], and ROTAVAC [Bharat Biotech, India]) require three doses. Each vaccine has different cost characteristics, and some have experienced supply shortages prompting a number of countries to switch to a different product [16, 17].

This study aims to estimate the cost-effectiveness and benefit-risk of ROTARIX, and to compare its continued use with other rotavirus vaccines.

## Methods

### Study design and model

We used version 1.7.01 of the UNIVAC (Universal Vaccine) decision-support model. UNIVAC is a static cohort model with a finely disaggregated age structure (weeks of age < 5 years) and can be used to assess the impact, cost-effectiveness, and benefit-risk of a range of different vaccines. It features a standardised and user-friendly Excel-based interface with a standard set of input steps and outputs. The model has been widely used by low- and middle-income countries (LMICs) to support decision-making for new vaccine introductions, including RV vaccines [18].

We ran two types of analysis:

i) cost-effectiveness and benefit-risk analysis of ROTARIX (1-dose vial) over a seven-year period (2018–2024) compared to no vaccination; and,
ii) cost-effectiveness and benefit-risk analysis of ROTARIX (1-dose vial), ROTASIIL (1-dose vial), ROTASIIL (2-dose vial), and ROTAVAC (5-dose vial) over a ten-year period (2025–2034). ROTATEQ was excluded from the comparison as it is not covered under Gavi’s financial support. Each product was compared to no rotavirus vaccination and each other.

In both analyses, we estimated the numbers of RVGE cases, outpatient visits, admissions, and deaths with and without RV vaccination. We also estimated potential excess intussusception cases and deaths using previously described methods [19]. Vaccine programme costs and healthcare costs were estimated throughout the first five years of life, and disability-adjusted life-years (DALYs) were calculated over the lifetimes of the target birth cohorts. The primary outcome measure was the discounted cost per DALY averted (from government and societal perspectives) compared to no vaccination. The benefit-risk of vaccination was represented by the estimated number of RVGE deaths prevented per one excess intussusception death.

Since 2018, the estimated annual gross domestic product (GDP) per capita in Afghanistan has rarely exceeded US$ 500, so we compared our estimates of the cost per DALY averted to a range of potential cost-effectiveness thresholds (CETs) between US$ 0 and US$ 500 [20, 21]. In compliance with WHO vaccine economic evaluation guidelines, we considered both government and societal perspectives, and all future costs and health benefits were presented at a discounted rate of 3% per year, and expressed in 2022 United States Dollars (US$) [22].

### Data collection and consensus building

Demographic projections were pre-populated in the model from the United Nations Population (UNPOP), providing population size by age/year, life expectancy at birth by age/year, and < 5 all-cause mortality by year. All other model inputs and their sources are shown in Tables 1 and 2.

**Table 1:** Input parameters for estimating the burden of rotavirus gastroenteritis (RVGE) in Afghanistan- 2018-2027

Parameter	Mid-point value	Low bound	High bound	Sources
Annual rate per 100,000 < 5 children				
RVGE non-severe cases	8224	6990	9458	[23]
RVGE non-severe RVGE visits	4367	3712	5022	[23, 24]
RVGE severe cases	1776	1510	2042	[4, 25]
RVGE severe visits	943	802	1085	[24, 25]
RVGE hospital admissions	444	377	511	[26] inflated by DTP 1 coverage in 2022 (73% as proxy of getting healthcare)
RVGE deaths	26	22	30	[27]
Intussusception cases	30	26	35	[4, 13, 28] inflated to account for those without access, using 2022 DTP 1 coverage (77%)
Intussusception hospital admissions	23	20	26	[13, 29]
Intussusception deaths	7	6	8	[13, 29]
Relative risk of intussusception				
After 1–7 days				
Dose 1	1.00	1.00	1.00	[13]
Dose 2	1.00	1.00	1.00	
After 8–21 days				
Dose 1	1.30	1.11	1.50	
Dose 2	1.00	1.00	1.00	
Age distribution of non-severe RVGE (%)				
<1 month	1%			[30]
<2 months	2%			
<3 months	6%			
<6 months	28%			
< 12 months	72%			
<24 months	96%			
<36 months	99%			
<48 months	100%			
<60 months	100%			
Age distribution of severe RVGE (%)				
<1 month	0%			[30]
<2 months	2%			
<3 months	7%			
<6 months	30%			
< 12 months	72%			
<24 months	94%			
<36 months	98%			
<48 months	100%			
<60 months	100%			
Intussusception age distribution**				
<1 month	0%			[13]
<2 months	0%			
<3 months	1%			
<6 months	30%			
< 12 months	70%			
<24 months	89%			
<36 months	95%			
<48 months	98%			
<60 months	100%			
DALY calculation				
Non-severe RVGE				
DALY weight	0.19	0.12	0.26	[31] (proxy: Moderate diarrhoea)
Duration of illness (days)	3	3	7	[32]
Severe RVGE				
DALY weight	0.25	0.16	0.35	[31] (proxy: Severe diarrhoea)
Duration of illness (days)	7	6	8	[32]
Intussusception				
DALY weight	0.32	0.22	0.44	[31] (Abdominopelvic problem, severe)
Duration of illness (days)	7	5	9	[13]
Vaccine effectiveness				
dose 1, initial VE	100%	45%*	100%	[12] VE and 95%CI Assume efficacy of 3 doses equal to 2 doses Fitted the observed effectiveness of each dose and time of administration to the post-licensure surveillance data Gamma waning curve (mean=10, alpha=3) reproduces VE of 93.8%, 73.1%, and 30.3% at 3, 6, and 12 months of follow-up respectively
dose 2, initial VE	100%	45%*	100%	
dose 3, initial VE	100%	45%*	100%	
Mean duration of VE after each dose (in months)	10	10	10	
Parameter 2 (alpha or shape)	3	3	3	
Vaccine coverage				
Dose 1				
2018	81%	69%	93%	[29], DTP1 as proxy for RV dose1 Aligned with observed coverage in post-licensure surveillance.
2019	75%	64%	86%	
2020	78%	66%	90%	
2021	74%	63%	85%	
2022	77%	65%	89%	
2023-2034	77%	65%	89%	
Dose 2				
2018	77%	65%	88%	[29] Average DTP 1 and 3, proxy for RV dose 2 Aligned with observed coverage in post-licensure surveillance.
2019	74%	62%	85%	
2020	74%	63%	85%	
2021	70%	60%	81%	
2022	73%	62%	84%	
2023-2034	73%	62%	84%	
Dose 3				
2018	72%	61%	83%	[29] 2022, DTP3, proxy for RV dose 2 Aligned with observed coverage in post-licensure surveillance.
2019	72%	61%	83%	
2020	70%	60%	81%	
2021	66%	56%	76%	
2022	69%	59%	79%	
2023-2034	69%	59%	79%	
Coverage timeliness				
Median age at dose 1, in weeks (IQR)	7	6	12	[24, 33]
Median age at dose 2, in weeks (IQR)	20	16	25	
Median age at dose 3, in weeks (IQR)	29	19	43	
% of doses administered by age				
Dose 1				
<1 month	0%			[33]
<2 months	67%			
<3 months	80%			
<6 months	90%			
< 12 months	95%			
<24 months	98%			
35-59 months	100%			
Dose 2				
<1 month	1%			[33]
<2 months	4%			
<3 months	41%			
<6 months	81%			
< 12 months	93%			
<24 months	97%			
34-59 months	100%			
Dose 3				
<1 month	0%			[33]
<2 months	0%			
<3 months	7%			
<6 months	77%			
< 12 months	96%			
<24 months	100%			
35-59 months	100%			
Vaccine price per dose (US$) with Gavi subsidy				
ROTARIX, 1-dose per vial, Liquid	$0.20			[15]
ROTASIIL, 1-dose per vial, liquid	$0.13			
ROTASIIL, 2-dose per vial, liquid	$0.13			
ROTAVAC, 5-dose per vial, liquid	$0.13			
Vaccine price per dose (US$) without Gavi subsidy				
ROTARIX, 1-dose per vial, Liquid	$ 2.36			
ROTASIIL, 1-dose per vial, liquid	$ 1.05			
ROTASIIL, 2-dose per vial, liquid	$ 0.80			
ROTAVAC, 5-dose per vial, liquid	$ 1.15			
International handling (% of vaccine price)	3.00%	2.55%	4.50%	[34]
International delivery (% of vaccine price)	5.00%	4.00%	7.50%	
Vaccine wastage rate				
ROTARIX, 1-dose, Liquid	5%	4%	8%	[35]
ROTASIIL, 1-dose, liquid	5%	4%	8%	
ROTASIIL, 2-dose, liquid	9%	8%	14%	
ROTAVAC, 5-dose, Liquid	15%	13%	23%	
Syringes wastage rate	5%	4%	8%	[35]
Health system delivery cost per dose (US$)	2.18	1.09	4.36	[36]
Additional health system cost per dose in the first year (2025) associated with switching from ROTARIX to ROTAVAC/ROTASIIL (US$)^§^	0.80	0.68	0.92	[17, 36] Average of switching cost reported in Palestine and Ghana studies Lower bound: Ghana: US$0.68 per dose Higher bound: Palestine: US$0.92 per dose

^§^ We used the mid-range between the switching costs reported by Palestine’s and Ghana’s studies divided by the number of doses to deliver in year 1. We used the low and high values for sensitivity analysis

**Table 2:** Input parameters for estimating health service costs in 2022 US$, Afghanistan

Parameter	Mid-point value	Low bound	High bound	Sources
Healthcare costs of RVGE				
Government perspective				
Non-severe outpatient visit (US$ 2022)	$5.04	$2.52	$10.08	[37]
Severe outpatient visit (US$ 2022)	$5.04	$2.52	$10.08	
Severe hospitalization (US$ 2022)	$17.56	$8.78	$35.12	
Societal perspective				
Non-Severe outpatient visit (US$ 2022)	$15.42	$7.71	$30.84	
Severe outpatient visit (US$ 2022)	$15.42	$7.71	$26.46	
Severe hospitalization (US$ 2022)	$38.05	$19.03	$76.10	
Healthcare costs of intussusception (IS)				
Government cost of IS hospitalization (US$ 2022)	$214.75	107.38	$429.50	[37, 38]
Societal cost of IS hospitalization (US$ 2022)	$234.38	117.19	$468.76	

To build consensus on model input parameters, we conducted consultation sessions with seven national experts between March and June 2024 during data collection and analysis. We engaged national experts in epidemiology, paediatric, immunization, health economics, and health system, representing stakeholders from the national expanded programme on immunization (EPI), WHO, UNICEF, and academia. This evaluation obtained approval from the Research Ethics Committee (ID: 29622, July 2023) of the London School of Hygiene and Tropical Medicine (LSHTM).

### RVGE disease burden

All disease input parameters are shown in Table 1. We estimated the incidence of severe RVGE cases in children by multiplying the WHO Eastern Mediterranean region estimate of the rate of severe AGE (5,972 per 100,000 per year, aged < 5 years) by the rotavirus fraction among severe AGE cases (29.74%) [25]. The rotavirus fraction was estimated using the mean of three estimates provided by Maternal and Child Epidemiology Estimation (MCEE), WHO/Centres for Disease Control and Prevention (WHO/CDC), and Global Burden of Disease (GBD) [4]. The incidence of non-severe RVGE cases was estimated by subtracting the severe RVGE rate from the overall RVGE case rate (10,000 per 100,000 per year, aged < 5 years) estimated in a global systematic review and meta-analysis by Bilcke et al. [23]. The rate of RVGE outpatient visits was calculated by multiplying the number of non-severe and severe RVGE cases by the proportion of children with diarrhoea seeking care (53.10%), as estimated from the Afghanistan Multiple Indicator Cluster Survey (MICS) 2022–2023 [24]. The rate of RVGE hospital admissions was estimated using pre-vaccination RVGE surveillance in two regions of Afghanistan, Central and Western [26]. To estimate the RVGE mortality rate we used the mean of estimates by MCEE, WHO/CDC and GBD [27].

The age distribution of severe RVGE (community cases, outpatient visits, admissions, and deaths) was estimated by week of age < 5 years using a parametric (Burr) distribution fitted to data from Afghanistan pre-vaccine surveillance (2013–2015) (Figure S1- panel a) [30]. In the absence of national data on the age distribution of non-severe RVGE (community cases and outpatient visits), we used estimates for Pakistan from a study by Hasso-Agopsowicz and colleagues [30] (Table 1).

For calculating DALYs, we used disability weights from Salomon et al. [31] and for duration of illness, we assumed 7 days for severe RVGE cases and 3 days for non-severe RVGE cases [32].

### RVGE healthcare cost

The cost per RVGE inpatient admission was taken from a systematic review of LMICs by Baral et al. which estimated the cost per gastroenteritis (GE) admission. The same source was used to estimate the cost per outpatient visit [37]. For the base case analysis, we included direct medical costs in the government perspective and the sum of direct medical, direct non-medical, and indirect costs in the societal perspective. We assumed that the cost per outpatient visit would be the same for severe and non-severe cases. Our analysis did not include costs for treatment given at home or through the informal sector (Table 2).

### Vaccination coverage, timeliness, and effectiveness

Observed RV vaccine coverage from post-licensure surveillance was very similar to the national administrative coverage reported in the WHO/UNICEF Estimates of National Immunization Coverage (WUENIC) for DTP1 and DTP3. Despite disruptions caused by the COVID-19 pandemic and political changes, the official administrative rotavirus vaccination coverage was reported to be stable from 2020 to 2022. We therefore used 2022 WUENIC estimates of DTP1 and DTP3 vaccine coverage as a proxy for RV1 and RV3 vaccine coverage and assumed this would remain consistent throughout our evaluation period [29]. Timeliness of vaccination was calculated by normalising coverage to 100%, representing the percentage of total coverage achieved by each age group up to 60 months. We incorporated real-world vaccine delays and timeliness (coverage by week of age) using data from MICS 2022, a recently nationally representing survey [24, 33] (Table 1).

We used estimates from a recent test-negative case-control study to approximate VE by time since dose administration using follow-up durations of 8 and 16 months for dose 1, and 7 and 15 months for dose 2 [12]. We fitted a parametric gamma curve to each dose, assuming VE would be very high shortly after dose administration and then fall to very low levels after around 18 months of follow-up (Figure S2). Estimates of VE and waning were very similar for doses 1 and 2, so we assumed the same waning rate for both.

Consistent with our previous analysis, we did not account for the indirect benefits of rotavirus vaccination, such as herd immunity, nor did we apply age restrictions to the vaccination schedule.

### Vaccine price and delivery cost

According to Gavi’s eligibility and transition policy version 4 (effective from January 2023) Afghanistan is classified as being in the initial self-financing phase [39]. In our base-case scenario, we applied a co-financing contribution of US$ 0.20 per dose for ROTARIX (2-doses) and US$ 0.13 per dose for ROTAVAC (3-doses) and ROTASIIL (3-doses). In scenario analysis, we ran a separate analysis assuming the government would pay the full price for ROTARIX (US$2.36 per dose for the 1-dose vial presentation), ROTASIIL (US$ 0.80 for the 2-dose vial presentation, and US$ 1.05 for the 1-dose vial presentation) and ROTAVAC ($US 1.15 per dose for the 5-dose vial presentation) [15]. We assumed prices would remain unchanged over the evaluation period (Table 1).

We applied wastage rates of 5%, 9%, and 15% for 1-dose, 2-dose and 5-dose vaccine presentations, respectively. These values were informed by reviewing the Comprehensive Multi-Year Plan (cMYP) 2021–2025 ([35]& Afghanistan EPI experts). A relatively high wastage rate of 15% for the 5-dose vaccine presentation has been derived from the current high wastage rates of the 1-dose and 2-dose vaccine presentations in the country. To calculate the cost of international handling and delivery, we applied 3% and 5% of the vaccine price, respectively [35]. The per dose cost of a safety bag with a capacity of 100 doses was estimated to be US$ 0.0056 [35]. No updated national data were available to estimate the health system delivery costs of rotavirus vaccination, so we used estimates for LMICs by Portnoy et, al (US$2.18 per dose in US$2022) [34] (Table 1). Due to limited data on the cost of switching products, we applied the average switching costs, which primarily cover the expenses of training, social mobilization, IEC materials, and stakeholder engagement, as reported by Palestine and Ghana, to the year of switching (2025) [17, 40]. We calculated the mid-range cost between Palestine and Ghana and divided it by the number of doses to be delivered in the first year with the 3-dose products (Table 1).

### Intussusception burden, risk, and costs

Intussusception (IS), invagination of a bowel segment causing blockage, is a rare but fatal medical condition if left untreated [5, 36]. In some (mostly high-income) settings rotavirus vaccination has been associated with a small excess risk of IS [5]. We estimated the potential number of excess IS cases, hospital admissions and deaths to account for the potential excess risk of IS in the 1–7 and 8–21 days following the first and second dose of RV vaccination. The post-licensure self-controlled case series (SCCS) study for ROTARIX in Afghanistan reported a small relative risk (RR = 1.3) in the 8–21 day period following administration of the first dose, so we included this risk in the base analysis [13].

Our estimates of the background rate of IS cases, hospital admissions, and mortality were obtained from post-licensure surveillance of children aged < 12 months. This was the best available data source given that no IS surveillance existed pre-vaccine, and the RR of vaccination was very minor and restricted to specific age windows post-vaccination. The admission rate < 12 months was rescaled to age < 5 years using estimates from a systematic review of the proportion of < 5 years IS hospital admissions that occurred by age 1 year in the WHO Eastern Mediterranean region [4, 13, 41]. The incidence rate of IS cases was derived by inflating the IS hospital admission rate to account for the percentage of children without access to care, using WUENIC estimates of the coverage of DTP1 in the year 2022 as a proxy for the maximum possible access to healthcare for severe conditions [13, 29] (Table 1). Estimates of the background rate of IS mortality were based on observed fatality rates among children < 1 year from post-licensure surveillance. These were rescaled to represent children < 5 years and inflated by DTP1 coverage to account for the percentage of children without access to care [13, 19]. The age distribution of all intussusception outcomes was calculated by week of age < 5 years using a parametric (Burr) distribution fitted to post-licensure surveillance data. (2018–2022) [13] (Figure S1- panel b).

Direct healthcare costs associated with intussusception treatment were included in the government perspective. These were estimated using the average cost per bed day in the National Hospitals Cost Analysis report, considering the average number of 5 days required for IS treatment [28]. From the societal perspective, we also included direct non-medical cost and indirect cost estimates from Baral’s study without accounting for any allowance for the cost of surgery [37] (Table 2).

### Uncertainty analysis

We conducted deterministic scenario analyses to assess the sensitivity of the cost-effectiveness results to changes in different combinations of parameter values. We applied -/+15% to lower and higher bound of base values to the base case values [38]. We considered the reported switch cost per dose of each country, Palestine and Ghana, to determine the lower and upper bounds of the vaccine delivery cost in the first year of the switch for the sensitivity analysis. We ran a scenario unfavourable to RV vaccination using the upper bound of the vaccine delivery cost per dose, a vaccine price without Gavi’s subsidy, the lower bounds of the disease burden rate, and the lower bounds of the healthcare costs. Although Gavi currently provides financial support for vaccine procurement in Afghanistan, its funding is designed to be time-limited, following a co-financing model where countries gradually take on a greater share of vaccine costs. Given the political and economic uncertainty in Afghanistan, there is no clear trajectory for the country’s transition within Gavi’s framework. Therefore, assessing the cost-effectiveness of rotavirus vaccination in a scenario without Gavi support is essential for long-term planning and sustainability considerations. We also ran scenarios favourable to the cost-effectiveness of RV vaccination by assuming “on-time” vaccine administration and using the upper bounds of the RVGE disease burden rates and healthcare costs, and lower bound of vaccine delivery costs. Lastly, in one of the one-way sensitivity analyses, we assumed no vaccine impact on non-severe RVGE. This assumption is influential because rotavirus vaccines have demonstrated higher efficacy and effectiveness against severe rotavirus gastroenteritis, while their impact on non-severe cases is more limited, as observed in both clinical trials and post-licensure studies. This reflects a conservative scenario, aligning with real-world data where vaccine effectiveness is primarily seen in reducing severe disease, hospitalizations, and mortality rather than preventing all rotavirus infections.

We ran a probabilistic sensitivity analysis (PSA) with 1000 Monte Carlo simulation runs. Within each run of the model, all parameters were simultaneously varied within their specified ranges. This was done to generate a 95% uncertainty interval around the central estimates of the cost per DALY averted. It was also done to determine the proportion of probabilistic runs with a cost-effectiveness ratio falling below CETs ranging from US$ 0 to US$ 500.

### Role of the funding source

The funder of the study had no involvement in the study design, data collection, data analysis, data interpretation, or report writing. The authors had full access to all the data in the study and had final responsibility for the decision in submit for publication.

## Results

We estimated that seven years (2018–2024) of ROTARIX use in Afghanistan prevented 1.72 million RVGE cases, 911,337 outpatient visits, 86,444 RVGE hospital admissions and 4,644 RVGE deaths (a 41% reduction), compared to a scenario with no RV vaccination. We estimated 1,493 RVGE deaths prevented for every one potential excess intussusception death (Table 3).

**Table 3:** Projected impact and cost-effectiveness of rotavirus vaccination in cohort vaccinated over period of 2018-2024 (DALY and costs discounted), government and society perspectives

Parameter	No vaccine	ROTARIX	
		With Gavi support	Without Gavi support
Lifetime costs and effects			
Non-Severe RVGE cases <5 yrs	3,886,826	2,516,352	2,516,352
Non-RVGE clinic visits <5 yrs	2,063,904	1,336,183	1,336,183
Severe RVGE cases <5 yrs	839,407	493,616	493,616
Severe RVGE clinic visits <5 yrs	445,725	262,110	262,110
Severe RVGE hospital admission <5 yrs	209,843	123,399	123,399
Severe RVGE deaths <5 yrs	11,270	6,626	6,626
Intussusception cases <5 yrs	14,179	14,193	14,193
Intussusception deaths <5 yrs	3,034	3,037	3,037
DALY averted (discounted*)	372,654	250,689	250,689
Vaccine programme costs (discounted*)	$0	$31,110,501	$62,760,329
Government healthcare costs (discounted*)	$16,257,205	$10,947,422	$10,947,422
Societal healthcare costs (discounted*)	$44,795,458	$28,900,879	$28,900,879
Differences (comparator = no vaccine)			
Non-Severe RVGE cases <5 yrs	-	1,370,474	1,370,474
Non-RVGE clinic visits <5 yrs	-	727,721	727,721
Severe RVGE cases <5 yrs	-	345,791	345,791
Severe RVGE clinic visits <5 yrs	-	183,615	183,615
Severe RVGE hospital admission <5 yrs	-	86,444	86,444
Severe RVGE deaths <5 yrs	-	4,644	4,644
Intussusception cases <5 yrs	-	-15^¥^	-15^¥^
Intussusception deaths <5 yrs	-	−3^¥^	−3^¥^
Percent reduction in severe RVGE deaths <5 yrs	-	41%	41%
Percent increase in Intussusception deaths <5 yrs	-	−0.10%	−0.10%
DALYs (discounted*)	-	121,965	121,965
Vaccine programme costs (discounted*)	-	$31,110,501	$62,760,329
Government healthcare costs (discounted*)		-$5,309,783	-$5,309,783
Societal healthcare costs (discounted*)	-	-$15,894,579	-$15,894,579
Cost (US$) per DALY averted (comparator = no vaccine)			
Governmental perspective			
Cost (discounted*)	-	$25,800,718	$57,450,546
DALYs averted (discounted*)	-	121,965	121,965
Cost per DALY averted (discounted*)	-	$212	$472
GDP per capital (2018)	-	$500	$500
Cost per DALY averted (discounted*) - % of GDP/capita	-	42.31	94.21
Societal perspective	-		
Cost (discounted*)	-	$15,215,921	$46,865,749
DALYs averted (discounted*)	-	121,965	121,965
Cost per DALY averted (discounted*)	-	$125	$386
GDP per capital (2018)	-	$500	$500
Cost per DALY averted (discounted*) - % of GDP/capita	-	24.95	76.85

*Future costs/effects were discounted at a rate of 3% per year

^¥^ Negative values indicate more cases or deaths with vaccination

With a heavily reduced vaccine dose cost (due to external donor support from Gavi) the estimated vaccine programme cost was US$31.11 million over seven years. Without Gavi support, this increased to US$ 62.76 million. We estimate that the cost of vaccination could be offset by US$ $5.31 million in RVGE treatment costs from the government perspective (or US$ 15.89 million from the societal perspective) (Table 3).

In the base case analysis, factoring in Gavi’s subsidy, the discounted cost per DALY averted was US$212 (0.42 times the national GDP per capita) from the government perspective and US$125 (0.25 times the national GDP per capita) from the societal perspective. However, when incorporating the full vaccine price without Gavi’s subsidy, the discounted cost per DALY averted increased to US$472 (0.94 times the national GDP per capita) from the government perspective and US$386 (0.67 times the national GDP per capita) from the societal perspective.

Scenario analysis showed that the cost-effectiveness results were most sensitive to the price per dose (i.e. whether or not Gavi financial support was included), the burden of RVGE disease and the vaccine delivery costs. With a reduced dose price (due to Gavi support), low vaccine delivery costs and high healthcare costs, the discounted cost per DALY averted was US$ 170 from the government’s perspective and US$ 71 from a societal perspective. Assuming no effect on non-severe RVGE, the estimated cost per DALY averted increases to US$ 242, compared to US$ 212 in the base case scenario from the government’s perspective. A similar difference was observed from the societal perspective. Additional details on various scenarios are provided in supplement Figure S3.

**Fig. 1:**
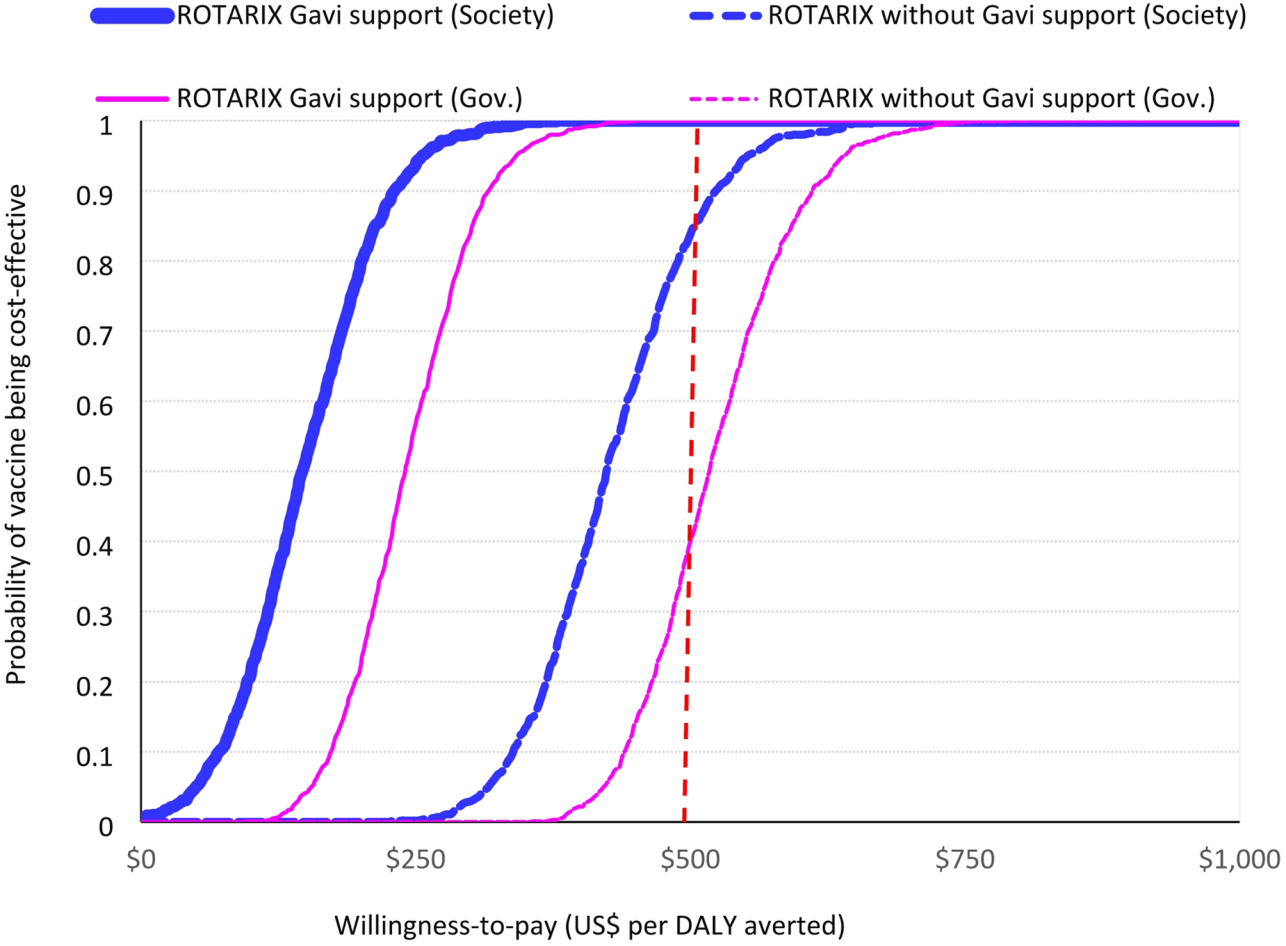
Probability that vaccination with ROTARIX is cost-effective at different level of willingness-to-pay (2018–2024). Probability that vaccination with ROTARIX is cost-effective at different willingness-to-pay thresholds from government and societal perspectives. The dashed vertical line is 1x GDP per capita (US$ 500)

ROTARIX had a 95% probability of being cost-effective when using a CET of US$250 (half the national GDP per capita). The full results of the probabilistic sensitivity analysis, including the cost-effectiveness plane and cost-effectiveness acceptability curve, are presented in Fig. 1 and supplement Figure S4 (panel a, b).

**Fig. 2:**
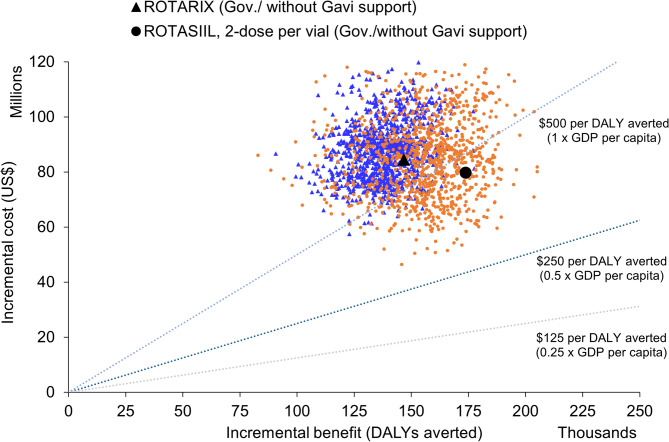
ROTARIX and ROTASIIL, 2-dose per vial without Gavi’s support and from government perspective. Probabilistic clouds showing the incremental cost (US$) and benefits (DALY averted) of rotavirus vaccine products compared to no vaccine, and each other without Gavi’s financial support in Afghanistan from *government* perspective, 2025-2034. ROTASIIL 2-dose per vial (in orange) without Gavi support had the most favourable cost-effectiveness (below 1x GDP per capita). The other three products namely ROTARIX 1-dose ROTASIIL 1-dose per vial, ROTAVAC 5-dose per vial had quite similar cost-effectiveness with higher incremental benefit but at the higher incremental costs compared to ROTASIIL 2-dose per vial. Their clouds overlapped, thus we presented only ROTASIIL 2-dose and ROTARIX on the plane. Under the probabilistic sensitivity analyses, we assumed a fixed *vaccine* price over the evaluation period. Thus, the probabilistic clouds would be very sensitive to changes in vaccine price

Over the period 2025–2034, ROTARIX was the most cost-effective product when Gavi-subsidised prices were used (Table 4, Figures S5 and S6). The estimated cost per DALY averted for continued use of ROTARIX was US$259 per DALY averted from a government perspective (and US$ 152 from a societal perspective) (Table 4). Without Gavi support, ROTASIIL (2-dose vial) dominates (Table 4; Fig. 2) and there is 35% probability that three-dose vaccines could be cost-effective at a CET of US$ 500 per DALY averted from a government perspective without Gavi’s support (Figure S6 panel a). With Gavi’s support from a societal perspective this probability increases to 95% (Figure S6 panel b).

**Table 4:** Economic evaluation of rotavirus vaccine products with and without Gavi subsidy in Afghanistan over the period 2025-2034^§^

	No vaccine	With Gavi subsidy				Without Gavi subsidy			
		ROTARIX, 1 dose vial, Liquid	ROTASIIL, 1 dose vial, Liquid	ROTASIIL, 2 dose vial, Liquid	ROTAVAC, 5 dose, liquid	ROTARIX, 1 dose vial, Liquid	ROTASIIL, 1 dose vial, Liquid	ROTASIIL, 2 dose vial, Liquid	ROTAVAC, 5 dose, liquid
Lifetime costs and effects									
Non-Severe RVGE cases <5 yrs	5,982,804	3,870,487	3,468,324	3,468,324	3,468,324	3,870,487	3,468,324	3,468,324	3,468,324
Non-RVGE clinic visits <5 yrs	3,176,869	2,055,229	1,841,680	1,841,680	1,841,680	2,055,229	1,841,680	1,841,680	1,841,680
Severe RVGE cases <5 yrs	1,292,059	759,107	661,324	661,324	661,324	759,107	661,324	661,324	661,324
Severe RVGE visits <5 yrs	686,083	403,086	351,163	351,163	351,163	403,086	351,163	351,163	351,163
Severe RVGE hospital admission <5 yrs	323,002	189,769	165,324	165,324	165,324	189,769	165,324	165,324	165,324
Severe RVGE deaths <5 yrs	13,813	8,116	7,070	7,070	7,070	8,116	7,070	7,070	7,070
Intussusception cases <5 yrs	21,824	21,847	21,847	21,847	21,847	21,847	21,847	21,847	21,847
Intussusception deaths <5 yrs	3,719	3,723	3,723	3,723	3,723	3,723	3,723	3,723	3,723
DALY (discounted*)	447,553	300,865	273,727	273,727	273,727	300,865	273,727	273,727	273,727
Vaccine programme costs (discounted)	$0	$45,789,790	$66,814,597	$67,002,040	$67,927,617	$92,373,387	$95,746,701	$88,998,319	$103,166,934
Government healthcare costs (discounted*)	$23,992,598	$16,150,212	$14,668,045	$14,668,045	$14,668,045	$16,150,212	$14,668,045	$14,668,045	$14,668,045
Societal healthcare costs (discounted)	$66,109,732	$42,633,813	$38,195,960	$38,195,960	$38,195,960	$42,633,813	$38,195,960	$38,195,960	$38,195,960
Differences (comparator = no vaccine)									
Non-Severe RVGE cases <5 yrs	..	2,112,317	2,514,480	2,514,480	2,514,480	2,112,317	2,514,480	2,514,480	2,514,480
Non-RVGE clinic visits <5 yrs	..	1,121,640	1,335,189	1,335,189	1,335,189	1,121,640	1,335,189	1,335,189	1,335,189
Severe RVGE cases <5 yrs	..	532,953	630,736	630,736	630,736	532,953	630,736	630,736	630,736
Severe RVGE visits <5 yrs	..	282,998	334,921	334,921	334,921	282,998	334,921	334,921	334,921
Severe RVGE hospital admission <5 yrs	..	133,233	157,677	157,677	157,677	133,233	157,677	157,677	157,677
Severe RVGE deaths <5 yrs	..	5,698	6,743	6,743	6,743	5,698	6,743	6,743	6,743
Intussusception cases <5 yrs	..	−23^¥^	−23^¥^	−23^¥^	−23^¥^	−23^¥^	−23^¥^	−23^¥^	−23^¥^
Intussusception deaths <5 yrs	..	−4^¥^	−4^¥^	−4^¥^	−4^¥^	−4^¥^	−4^¥^	−4^¥^	−4^¥^
Percent reduction in severe RVGE deaths <5 yrs	..	41.25%	48.82%	48.82%	48.82%	41.25%	48.82%	48.82%	48.82%
Percent increase in Intussusception deaths <5 yrs	..	0.10%	0.10%	0.10%	0.10%	0.10%	0.10%	0.10%	0.10%
DALYs averted (discounted*)	..	146,689	173,826	173,826	173,826	146,689	173,826	173,826	173,826
Vaccine programme costs (discounted*)	..	$45,789,790	$66,814,597	$67,002,040	$67,927,617	$92,373,387	$95,746,701	$88,998,319	$103,166,934
Government healthcare costs (discounted*)	..	$7,842,387	$9,324,553	$9,324,553	$9,324,553	$7,842,387	$9,324,553	$9,324,553	$9,324,553
Societal healthcare costs (discounted*)	..	$23,475,918	$27,913,772	$27,913,772	27,913,772	$23,475,918	$27,913,772	$27,913,772	$27,913,772
Cost (US$) per DALY averted (comparator = no vaccine) (Government perspective)									
Governmental perspective									
Cost (discounted*)	..	$37,947,404	$57,490,044	$57,677,487	$58,603,063	$84,531,000	$86,422,147	$79,673,765	$93,842,381
DALYs averted (discounted*)	..	146,689	173,826	173,826	173,826	146,689	173,826	173,826	173,826
Cost per DALY averted (discounted*)	..	$259	$331	$332	$337	$576	$497	$458	$540
Proportion of the GDP per capita (%)		52%	66%	66%	67%	115%	99%	92%	108%
Societal perspective									
Cost (discounted*)	..	$22,313,872	$38,900,825	$39,088,268	$40,013,845	$68,897,468	$67,832,929	$61,084,547	75,253,163
DALYs averted (discounted*)	..	146,689	173,826	173,826	173,826	146,689	$173,826	$173,826	$173,826
Cost per DALY averted (discounted*)	..	$152	$224	$225	$230	$470	$390	$351	$433
Proportion of the GDP per capita (%)	..	30%	45%	45%	45%	94%	78%	70%	87%

		ROTARIX 1st option	ROTASIIL1 2nd option	ROTASIIL2 3rd option	ROTAVAC 4th option	ROTARIX 2nd option	ROTASIIL1 3rd option	ROTASIIL2 1st option	ROTAVAC 4th option
Cost (US$) per DALY averted (incremental analysis)									
Government perspective (US$)	..								
Costs (discounted*)	..	$37,947,404	$19,542,640	Dominated by ROTASIIL1	Dominated by ROTASIIL1	Dominated by ROTASIIL2	Dominated by ROTASIIL2	$79,673,765	Dominated by ROTASIIL2
DALY averted (discounted*)	..	146,689	27,138					173,826	
Cost per DALY averted (discounted*)	..	$259	$720					$458	
Societal perspective (US$)									
Costs (discounted*)	..	$22,313,872	$16,586,953					$61,084,547	
DALY averted (discounted*)	..	146,689	27,138					$173,826	
Cost per DALY averted (discounted*)	..	$152	$611					$351	

^§^Under the assumption of all products (3 doses vs 2 doses) have the same vaccine effectiveness

*Future costs/effects were discounted at a rate of 3% per year

^¥^ negative values indicate more cases or deaths with vaccination

In scenario analysis, the choice of product (product with the lowest cost per DALY averted) was most sensitive to the dose price, wastage rate, health system delivery cost, and assumptions about whether 3-dose vaccines would have the same or slightly higher impact than the 2-dose vaccine (ROTARIX) (Table 4).

## Discussion

We have updated our previous estimates of cost-effectiveness using real-world post-licensure national data and updated RVGE disease burden estimates. Our updated analysis shows that over a seven-year period (2018–2024), ROTARIX had an important public health impact, preventing around 12,000 hospital admissions and 650 deaths each year. Our analysis also estimates a favourable ratio of benefits to risks. We estimated important economic benefits, with ROTARIX averting up to US$5.31 million and US$15.89 million in RVGE treatment costs from government and societal perspectives, respectively. Assuming a heavily discounted dose price due to support from Gavi, we estimated that it cost US$212 and US$125 to prevent each DALY, from a government and societal perspective, respectively. This is less favourable than our previous analysis (US$31 and US$29 respectively), primarily because our previous analysis assumed higher rates of RVGE mortality in the absence of vaccination. Additionally, cost values were adjusted to reflect US dollars as of 2022, a period of significant inflation since 2018. Despite these increases, our current figures are consistent with existing literature; a meta-regression analysis reported a mean incremental cost-effectiveness ratio (ICER) of US$225 per DALY for Gavi-eligible countries [9].

Afghanistan has faced significant economic hurdles since the Taliban’s takeover in August 2021. The resulting economic downturn has not only strained the government’s ability to finance health interventions but has also reduced the population’s capacity to afford healthcare services. The country’s ranking has declined from 153 in 2022 to 181 in 2023–2024 out of 193 countries, based on composite human development indices [42]. Beyond financial constraints, the healthcare system’s absorption capacity has also been impacted, raising concerns about the ability to fully utilize available resources. Operational challenges, reduced healthcare access for women, workforce shortages, and shifting donor priorities, have affected the implementation and uptake of immunization programmes. Additionally, economic hardship may have contributed to lower vaccine demand and access, as families prioritize immediate survival needs over preventive health measures. Additionally, the GDP per capita, often used as a basis for interpreting CETs, has seen a 12–62% decline from US$ 562 in 2017 to date. Given this economic instability, we applied a range of CETs from $0- US$ 500 to account for different affordability scenarios. Our most favourable analysis suggests a cost-effectiveness ratio of 0.25 times the national GDP per capita, increasing to 0.94 times under assumptions least favourable to vaccination. Pichon-Rivere and colleagues recently recommended a CET of 0.65 time the national GDP per capita in Afghanistan using a method based on current levels of healthcare expenditure [43]. This suggests that RV vaccination is likely to remain cost-effective from a government perspective while it continues to benefit from the favourable assumption of Gavi subsidised prices. This seems likely given the recent political and economic disruption and increased need for donor assistance in Afghanistan. Looking ahead to the period 2025–2034, ROTARIX is projected to remain the dominant product (from a Government perspective), assuming continued Gavi support.

Affordability is an important consideration. With substantial external financial support from Gavi, the net cost to the Afghan government’s vaccine programme was estimated to be US$ 4.44 million annually, accounting for 5.5% of the annual EPI programme budget and around 0.13% of the total national health expenditure. Without Gavi support, the vaccine programme cost would surpass US$ 8.96 million per year.

We estimated a substantial health and economic impact of vaccination. Similar findings have been reported in other low-income countries from both government and societal perspectives [17, 19, 40, 42, 44, 45]. Data from the five post-licensure surveillance sites indicate a decline in rotavirus positivity among admitted RVGE cases from 51% in the pre-vaccine period (2013–2015) to 39% in the post-vaccine period (2019–2021), representing a 39% reduction [46]. We estimated a 41% reduction in RVGE hospitalizations per birth cohort from 2018 to 2024, closely aligning with the 39% decline in RVGE hospital admissions observed in the post-licensure evaluation. Historically, rotavirus vaccination has shown a lower performance in LMICs compared to high-income countries. Opportunities to increase impact might involve introducing an additional dose to mitigate the effects of waning vaccine protection. We estimate this has the potential to increase health impact by around 8% (41% for 2-dose vs. 49% for 3-dose vaccines).

We acknowledge the uncertainty in our estimates of mortality reduction, particularly given that our empirical endpoint for vaccine effectiveness was severe RVGE rather than direct mortality outcomes. While rotavirus vaccination has been shown to reduce severe gastroenteritis cases, the extent to which this translates into mortality reduction depends on several factors, including vaccination coverage, healthcare access, and the vulnerability of high-risk populations.

One key uncertainty is whether the children most at risk of RVGE-related mortality, typically those with limited access to healthcare, malnutrition, or underlying health conditions, are adequately reached and protected by vaccination. If vaccine coverage is lower among these high-risk groups, the expected mortality reduction may be overestimated.

To reduce this uncertainty, future research should consider evaluation of vaccine coverage disparities among high-risk and marginalized population, conducting post-introduction mortality studies, and exploring integrated health and nutrition interventions to maximize impact among the most vulnerable children.

Our analysis reaffirms the substantial benefits of RV vaccination compared to the potential excess risk of intussusception. We estimated 1,493 RVGE deaths prevented for every one potential excess intussusception death. Recent modelled estimates of benefit-risk for 135 LMICs reported a ratio of 1,503:1 [19]. We also ran a scenario with age restrictions applied (first dose < 15 weeks, last dose < 32 weeks). This adjustment reduced the vaccine’s impact to 25%, but the benefit-risk ratio subsequently improved to 2,790:1 because fewer doses were administered during the peak age of intussusception. More favourable benefit-risk ratio was reported in another modelled study when age restrictions were applied 2,385:1 [19].

The regime change in Afghanistan has broader implications beyond financial constraints, particularly in terms of vaccine equity and access. While our manuscript primarily focuses on the economic challenges, we recognize that restrictions on women’s mobility may pose additional barriers to immunization coverage, especially in settings where mothers are primary caregivers responsible for taking children to vaccination services.

Our analysis has some limitations. Our estimates of the burden of RVGE, vaccine effectiveness, and intussusception subject to uncertainty. One key challenge is the ambiguity surrounding the catchment population size. The last official census in the country was conducted in 1979, leaving a wide range of possible population estimates. To address this, we applied lower and upper bounds and used international estimates for some of the inputs. Additionally, we incorporated uncertainty analyses to reflect the variability in disease burden estimates and healthcare costs. Our estimates of the benefits of rotavirus vaccination may be underestimated because of the static nature of the UNIVAC model which does not capture possible herd immunity effects. Studies from other low-income settings suggest that herd immunity could reduce overall RVGE cases and hospitalizations by an additional 10–30%, which is not accounted for in our cost-effectiveness estimates [47]. Using a dynamic model in this context would introduce its own challenges e.g. it would require uncertain input parameters including social contact patterns and the duration of immunity from natural infections. The primary aim of this study is to support vaccine decision-making. CEA studies using dynamic models have reported lower costs per DALY averted compared to static models [48, 49] but these models do not account for potential clustering of rotavirus vaccination coverage in lower-risk populations. As a result, we do not expect this to fundamentally alter our conclusions regarding the cost-effectiveness of rotavirus vaccination in Afghanistan. Importantly, in this study we were able to use real-world data on VE from post-licensure surveillance and our estimates of the direct effects of vaccination closely replicated the observed impact.

## Conclusion

Our study underscores the importance of sustaining the use of rotavirus vaccination in Afghanistan. Future research should focus on enhancing vaccine delivery efficiency and addressing barriers to vaccine uptake, thereby increasing the public health impact of rotavirus vaccination.

## Supplementary Information

Supplementary Material 1.

## Data Availability

No datasets were generated or analysed during the current study.

## Abbreviations

AGE: Acute Gastroenteritis
CDC: Centers for Disease Control and Prevention
CEA: Cost-Effectiveness Analysis
CETs: Cost-Effectiveness Thresholds
cMYP: Comprehensive Multi-Year Plan
DALY: Disability-Adjusted Life Year
DTP1: Diphtheria, Tetanus, and Pertussis Dose 1
DTP3: Diphtheria, Tetanus, and Pertussis Dose 3
EPI: Expanded Programme on Immunization
GDP: Gross Domestic Product
GE: Gastroenteritis
GBD: Global Burden of Disease
IEC: Information, Education, Communication
ICER: Incremental Cost-Effectiveness Ratio
LMIC: Low- and Middle-Income Countries
LSHTM: London School of Hygiene and Tropical Medicine
MCEE: Maternal and Child Epidemiology Estimation
MICS: Multiple Indicator Cluster Survey
MoPH: Ministry of Public Health
NITAG: National Immunization Technical Advisory Group
PSA: Probabilistic Sensitivity Analysis
RV: Rotavirus
RVGE: Rotavirus Gastroenteritis
SCCS: Self-Controlled Case Series
UNIVAC: Universal Vaccine
VE: Vaccine Effectiveness
WHO: World Health Organization
WUENIC: WHO/UNICEF Estimates of National Immunization Coverage
