# Supplementary material for: Cost-effectiveness and benefit-risk of rotavirus vaccination in Afghanistan: a modelling analysis informed by post-licensure surveillance
Source: BMC Health Serv Res. 2025 Jul 4;25:926. doi: 10.1186/s12913-025-12885-5 (PMC12232007; doi:10.1186/s12913-025-12885-5)
Supplement: Supplementary file 1 — Supplementary Material 1. [file 12913_2025_12885_MOESM1_ESM.docx]

**Cost-effectiveness and benefit-risk of rotavirus vaccination in Afghanistan: a modelling analysis informed by post-licensure surveillance**

**Supplements tables and figures**

**Figure S1 panel a. Age fitting (to weekly age bands), RVGE cases pre-licensure surveillance, Afghanistan 2013-2015**


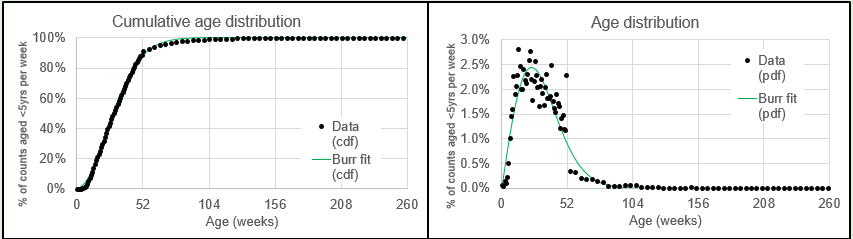


**Caption:** The left graph shows the cumulative age distribution of RVGE admission cases from pre-vaccine surveillance from two sites between 2013 and 2015. The right graph presents the Burr fitted age distribution curve of RVGE admission cases.

**Figure S1 panel b. Age fitting (to weekly age bands), Intussusception cases, post-licensure surveillance, Afghanistan 2018-2022**


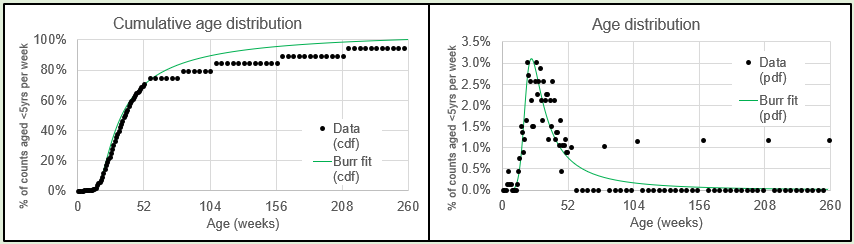


**Caption:** The left graph shows the cumulative age distribution of intussusception cases from post-vaccine surveillance from four sites between 2018 and 2022. The right graph presents Burr fitted age distribution curve of intussusception cases.

Figure S2 Approximating efficacy of rotavirus vaccination by time since dose administration in Afghanistan using data from a test-negative case control study*

*VE=100%; Mean efficacy duration= 10 months; Alpha or shape= 3

**Figure S3 Deterministic scenario analysis incremental cost-effectiveness ratio (US$ per DALY averted) of ROTARIX**

**Figure S3 Caption.**  Deterministic scenario analysis incremental cost-effectiveness ratio (US$ per DALY averted) of ROTARIX, compared to no vaccination. Discounted cost per DALY averted (US$) for various scenarios plotted against Gross Domestic Products (GDP) per capita of US$ 500 for the period 2018-2024. This series of analyses enabled us to pinpoint the most influential economic parameters. The lowest projected discounted cost per DALY averted was observed when Gavi provided financial support, maintaining low vaccine delivery costs while healthcare costs were at the upper bound.

**Figure S4 panel a. Probabilistic clouds of the incremental cost (US$) and benefits (DALY averted) of ROTARIX compared to no vaccine from societal perspectives, 2018-2024.**


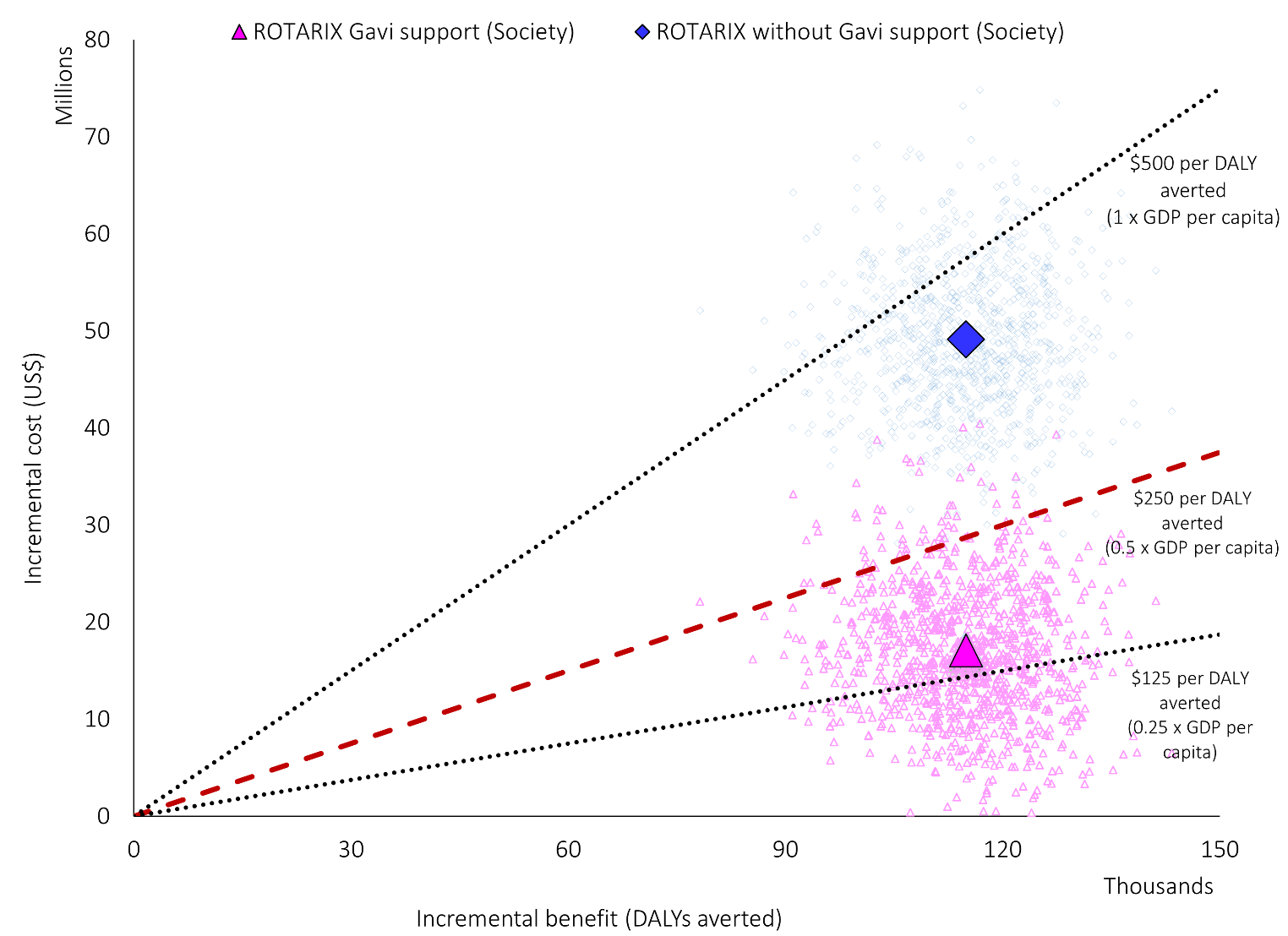


**Caption.** Probabilistic clouds showing the incremental cost (US$) and benefits (DALY averted) of ROTARIX compared to no vaccine with and without Gavi’s financial support in Afghanistan from societal perspectives, 2018-2024. ROTARIX with Gavi support [purple] and ROTARIX without Gavi support [blue]. ROTARIX with Gavi’s support would be cost-effective at the threshold of 1 x GDP per capita (US$500) from **societal perspective**. under the probabilistic sensitivity analyses we assumed a fixed price vaccine over the evaluation period. Thus, the probabilistic clouds would be very sensitive to changes in vaccine price.

**Figure S4 panel b. Probabilistic clouds of the incremental cost (US$) and benefits (DALY averted) of ROTARIX compared to no vaccine from government perspectives, 2018-2024.**


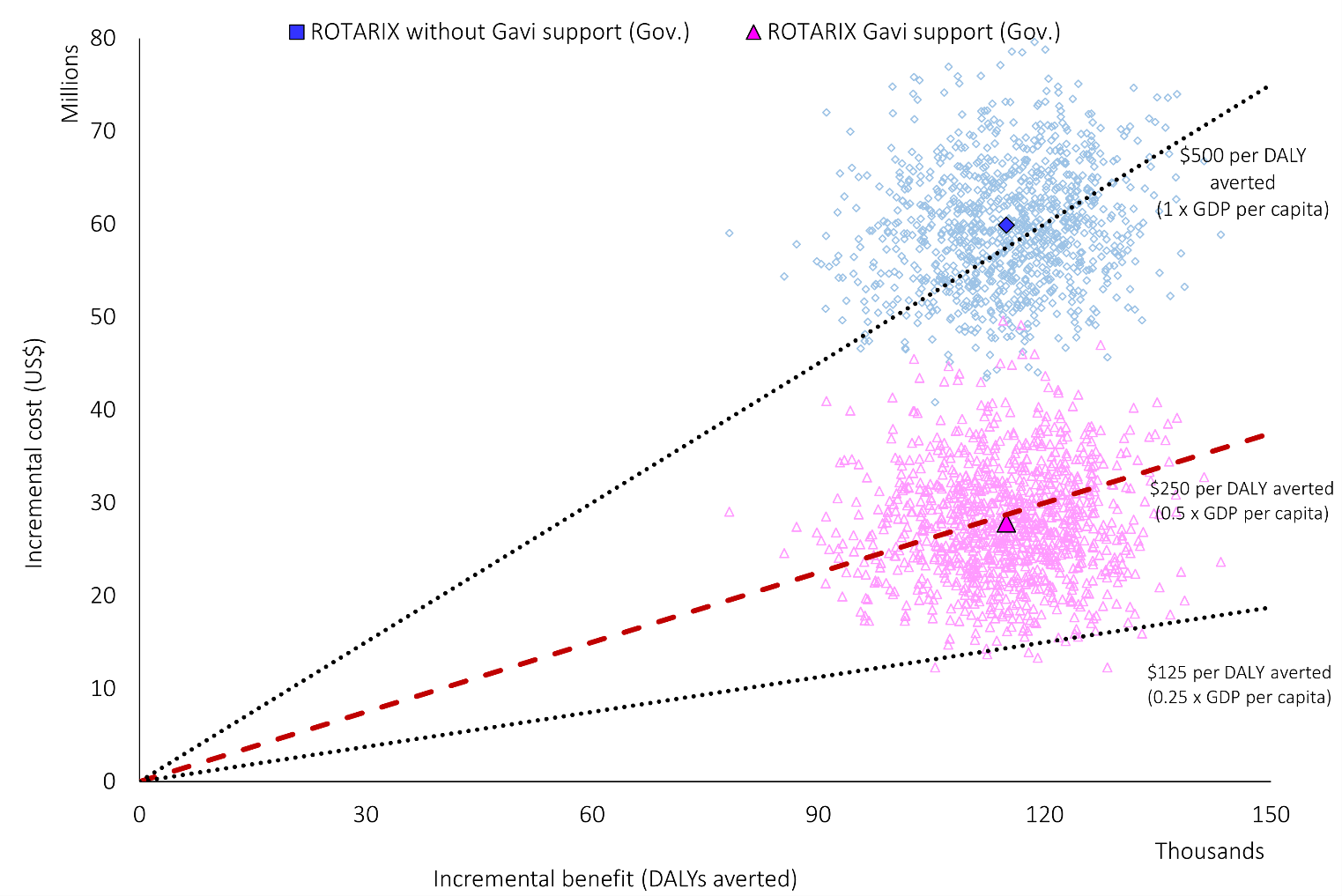


**Caption figure S4 panel b**. Probabilistic clouds showing the incremental cost (US$) and benefits (DALY averted) of ROTARIX compared to no vaccine with and without Gavi’s financial support in Afghanistan from government perspectives, 2018-2024. ROTARIX with Gavi support [purple] and ROTARIX without Gavi support [blue]. ROTARIX with Gavi’s support would be cost-effective at the threshold of slightly at 1 x GDP per capita (US$500) from **government perspective**. Under the probabilistic sensitivity analyses we assumed a fixed price vaccine over the evaluation period. Thus, the probabilistic clouds would be very sensitive to changes in vaccine price.

**Figure S5 ROTARIX, ROTASIIL, 2-dose per vial, and ROTAVAC, 5-dose per vial, with Gavi’s support and from society perspective**


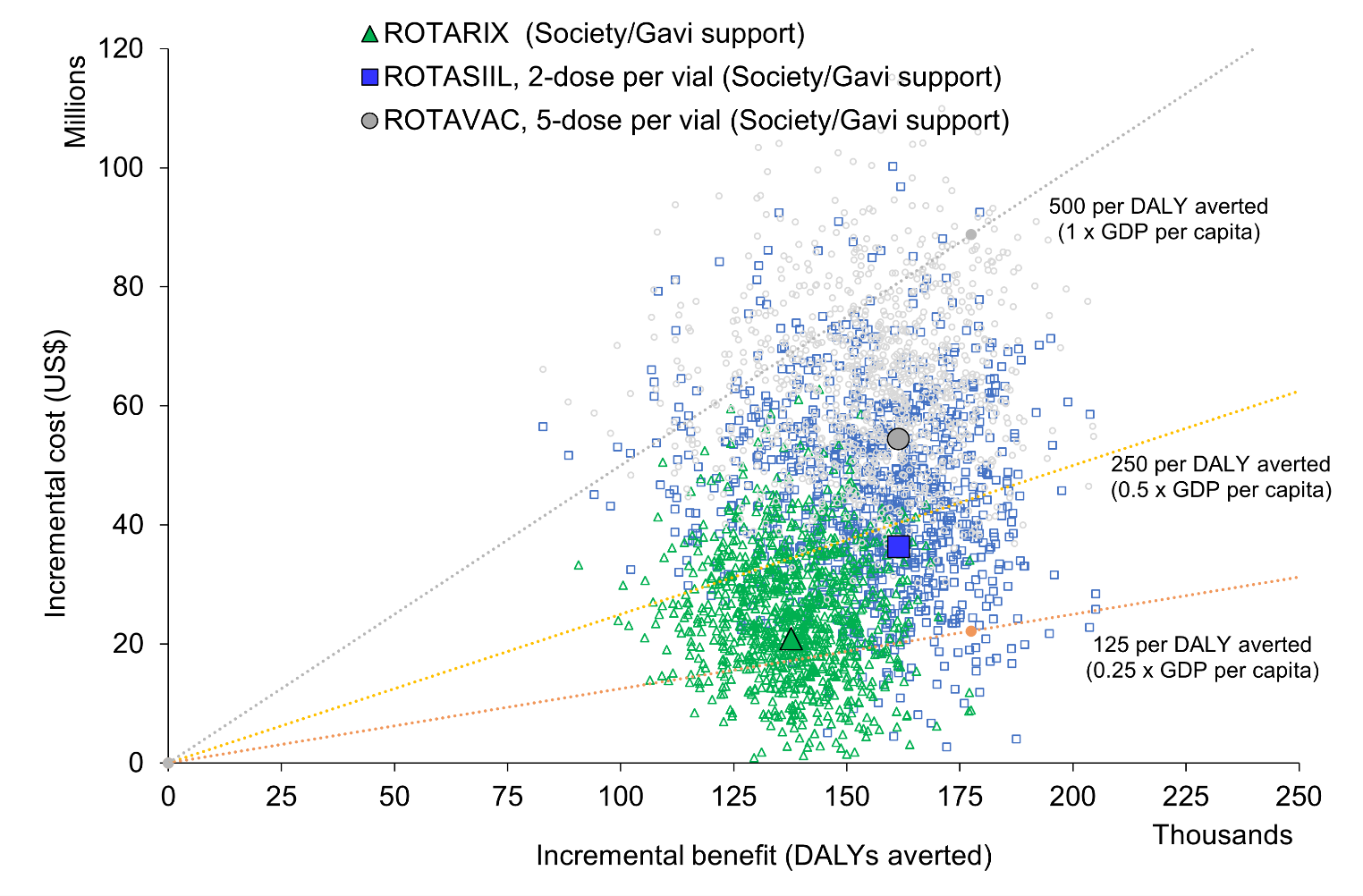


**Caption figure S5**. Probabilistic clouds showing the incremental cost (US$) and benefits (DALY averted) of rotavirus vaccine products compared to no vaccine, and each other with Gavi’s financial support in Afghanistan from *societal* perspective, 2025-2034. ROTARIX (in green) with Gavi support had the most favourable cost-effectiveness (at 0.5x GDP per capita*.* The other three products namely ROTASIIL 1-dose ROTASIIL 2-dose per vial, ROTAVAC 5-dose per vial had quite similar cost-effectiveness with higher incremental benefit at the higher incremental costs compared to ROTARIX. We dropped ROTASIIL 1-dose per vial because it completely overlapped ROTASIIL 2-dose per vial. Under the probabilistic sensitivity analyses, we assumed a fixed vaccine price over the evaluation period. Thus, the probabilistic clouds would be very sensitive to changes in vaccine price.

**Figure S6 panel a. Willingness to pay plot of rotavirus vaccine products from government perspective without Gavi support**

**Figure 6S *panel a caption.*** Probability that vaccination with four rotavirus vaccine products, ROTARIX, ROTASIIL (1-dose per vial and 2-dose per vial), and ROTAVAC would be cost-effective at different willingness-to-pay thresholds from societal perspective without Gavi’s subsidy. The dotted vertical line is 1x GDP per capita (US$*500*)

**Figure S6 panel b. Willingness to pay plot of rotavirus vaccine products from societal perspective, with Gavi support**

**Figure 6S *panel b caption.*** Probability that vaccination with four rotavirus vaccine products, ROTARIX, ROTASIIL, and ROTAVAC that would be cost-effective at different willingness-to-pay thresholds from societal perspective with Gavi’s subsidy. The dotted vertical line is 1x GDP per capita (US$*500*)
